# Clinical care standards for the management of low back pain: a scoping review

**DOI:** 10.1007/s00296-024-05543-2

**Published:** 2024-02-29

**Authors:** Gabriel S. Alves, Gustavo E. Z. Vera, Chris G. Maher, Giovanni E. Ferreira, Gustavo C. Machado, Rachelle Buchbinder, Rafael Z. Pinto, Crystian B. Oliveira

**Affiliations:** 1 Faculty of Medicine, University of Western São Paulo (UNOESTE), Presidente Prudente, Sao Paulo, Brazil; 2 Institute for Musculoskeletal Health, The University of Sydney, Royal Prince Alfred Hospital, Sydney Local Health District, Level 10N, King George V Building, Missenden Road, P. O. Box M179, Camperdown, 2050 Australia; 3 Sydney School of Public Health, Faculty of Medicine and Health, The University of Sydney, Sydney, NSW Australia; 4 Musculoskeletal Health and Wiser Health Care Units, School of Public Health and Preventive Medicine, Monash University, Melbourne, Australia; 5 Department of Physical Therapy, Universidade Federal de Minas Gerais (UFMG), Belo Horizonte, Minas Gerais, Brazil

**Keywords:** Low back pain, Clinical care standards, Quality of health care

## Abstract

The objective of this study is to compare and contrast the quality statements and quality indicators across clinical care standards for low back pain. Searches were performed in Medline, guideline databases, and Google searches to identify clinical care standards for the management of low back pain targeting a multidisciplinary audience. Two independent reviewers reviewed the search results and extracted relevant information from the clinical care standards. We compared the quality statements and indicators of the clinical care standards to identify the consistent messages and the discrepancies between them. Three national clinical care standards from Australia, Canada, and the United Kingdom were included. They provided from 6 to 8 quality statements and from 12 to 18 quality indicators. The three standards provide consistent recommendations in the quality statements related to imaging, and patient education/advice and self-management. In addition, the Canadian and Australian standards also provide consistent recommendations regarding comprehensive assessment, psychological support, and review and patient referral. However, the three clinical care standards differ in the statements related to psychological assessment, opioid analgesics, non-opioid analgesics, and non-pharmacological therapies. The three national clinical care standards provide consistent recommendations on imaging and patient education/advice, self-management of the condition, and two standards (Canadian and Australian) agree on recommendations regarding comprehensive assessment, psychological support, and review and patient referral. The standards differ in the quality statements related to psychological assessment, opioid prescription, non-opioid analgesics, and non-pharmacological therapies.

**Supplementary Information:**

The online version contains supplementary material available at 10.1007/s00296-024-05543-2.

## Background

Low back pain is the top contributor to global years lived with disability [1], and imposes massive healthcare costs worldwide [2, 3]. For example, the annual cost of healthcare for low back pain and neck pain in the United States was US$134.5 billion in 2016—the highest healthcare spend across all conditions [4]. A substantial contributor to the high expenditure and poor outcomes seen with low back pain is that many patients miss out on guideline-endorsed care and/or receive guideline-discordant care [5]. For example, routine imaging is discouraged in guidelines, yet nearly one-third of patients with non-specific low back pain are referred for lumbar imaging [6].

Many clinical practice guidelines for the management of low back pain have been published in the last decade [7]. Recently, organisations have developed documents to help implement the care that should be offered to patients considering the best available evidence [8]. These documents are called ‘clinical care standards’ or ‘quality standards of care’ and include a brief list of “*quality statements*” that describe the key aspects of care that should be provided [8]. The clinical care standards are typically proposed in high priority areas where there is unwarranted variation between evidence and clinical practice [9, 10].

There are differences between the structure of clinical practice guidelines and clinical care standards. First, while clinical practice guidelines provide evidence-based recommendations, the clinical care standards focus on the implementation and evaluation of these recommendations in health care settings. Second, clinical practice guidelines contain comprehensive information on management, whereas clinical care standards focus on a smaller number of critical areas for improvement of the quality of health care. A third difference is that clinical care standards provide “*quality indicators*” that can be used to monitor the uptake of standards.

Clinical care standards seem a promising approach for the implementation of evidence-based recommendations in health care settings. Recently, clinical care standards for low back pain targeting multidisciplinary audiences have been developed in several countries. We conducted a scoping review to compare and contrast the existing clinical care standards for low back pain. In particular, we (i) examined the quality statements across the standards and their accompanying content to identify discrepancies, inconsistencies, and unclear messages, and (ii) compared the quality indicators and investigated potential problems regarding feasibility, including the data sources suggested to allow monitoring of care quality. Investigating discrepancies and similarities between the clinical care standards may help to better understand these documents and guide the development of new clinical care standards for the management of low back pain.

## Methods

This is a scoping review evaluating all documents specified as clinical care standards or quality standards of care, including recommendations along with indicators for the diagnosis and treatment of low back pain. We followed the recommendations of the Preferred Reporting Items for Systematic reviews and Meta-Analyses for Scoping Reviews (PRISMA-ScR) [11].

### Searches

Literature searches were performed in the following databases on 18th July 2023: MEDLINE via OVID and Google (keywords: combination of low back pain AND ("care standards" or "quality standards")), International Guidelines Library (https://g-i-n.net/international-guidelines-library; keyword: low back pain), and National Institute for Health and Clinical Excellence (NICE) (www.nice.org.uk; keyword: low back pain). We also performed searches on Google using a combination of low back pain AND "care standards" or "quality standards". Two independent reviewers selected the potentially eligible reports, and a third reviewer was available to resolve any disagreements.

### Eligibility criteria

Clinical care standards for the management of low back pain were eligible for this review. To be considered eligible, the clinical care standards should include statements and indicators related to the diagnosis and treatment of non-specific low back pain, targeted at a multidisciplinary primary healthcare audience. We excluded clinical practice guidelines, clinical care standards targeting a specific profession or a broader health population (e.g., musculoskeletal disorders), or other documents including only quality indicators.

### Data extraction and synthesis

Two independent authors performed standardised data extraction, considering the following information: development methods (e.g., target audience, evidence source, working group, dissemination and implementation, and update time) as well as statements and indicators for diagnosis and treatment. The development methods, statements, and indicators were described in a table and compared to identify the agreement and discrepancies between the clinical care standards.

## Results

The searches in the electronic databases, guidelines databases, and Google retrieved 579 documents. After screening the titles and abstracts, eight potentially eligible documents were selected for the full-text assessment. Of these, two documents were excluded, because they were developed for a specific profession (e.g., chiropractors), and three were specified as clinical practice guidelines without providing statements and indicators. Finally, three clinical care standards were included in this review [12–14].

### General aspects of clinical care standards

The 2019 Canadian clinical care standard [12], developed by Health Quality Ontario, included 7 quality statements and 13 indicators for acute low back pain (i.e., first episode or recurrent episodes lasting less than 12 weeks). The 2017 UK clinical care standard [13], developed by the National Institute for Health and Care Excellence (NICE), has 6 statements and 18 indicators and specifies whether they apply to acute low back pain, chronic low back pain or sciatica. The 2022 Australian clinical care standard [14], developed by the Australian Commission on Safety and Quality in Health Care, has 8 statements and 12 indicators for people with low back pain presenting with a new acute episode. Table 1 details the development methods of the three clinical care standards.

**Table 1:** Development methods of UK, Canadian, and Australian clinical care standards

Topic	Clinical care standards
Target audience	• UK: NHS services, public health services and social care • Canadian: Primary care and community-based care, although it could be applied to all settings • Australian: Primary care and emergency departments but applies to all healthcare settings and clinical units
Evidence source	• UK: NICE guideline • Canadian: Several practice guidelines and supporting references • Australian: Several practice guidelines and supporting references
Working group	• UK: Standing Committee working across quality standard topics and specialist committee members which included a nurse, two general practitioners, a therapist, and a pain consultant • Canadian: Advisory committee membership included health care professionals (i.e., family physicians, spine surgeons, nurses, chiropractors, physiotherapists, psychologists), an associate professor, patients, caregivers, and others with lived experience • Australian: independent experts, consumers and clinicians from different areas of expertise (such as physiotherapists, chiropractors, rheumatologists, psychologists, emergency physicians, and others)
Stakeholders involvement	• UK: The consultation of stakeholders (i.e., national patient, service user, healthcare professional and academic organisations and commercial organisations) to identify key areas for improvement, provide feedback on the appropriateness of the formulated quality statements, and support the dissemination of the clinical care standard • Canadian: The participation of stakeholders to inform the development and quality of the recommendations and implementation/dissemination phase • Australian: The clinical care standard was opened for public consultation
Dissemination and implementation	• UK: Provide support to key audiences to maximise the uptake of guidance and quality standards, support tools for commissioning, service improvement and education and learning, engage with national bodies and local organisations to support the use and review of quality standards and facilitate shared learning; feedback on changes in the areas for quality of improvement • Canadian: To identify stakeholders to engage in dissemination and implementation planning. Target audiences for quality standards include health system partners, clinical leaders, administrators, advocacy organisations, community partners, patients, and caregivers. A toolkit is developed and may include a gap assessment tool, a barrier assessment tool, and general resources on change management. Dissemination is based on a communication plan that uses social media, traditional media, newsletters, webinars, and other channels to share updates, educate, and create awareness of the quality standard. Decision support tools, audit and feedback, educational materials and education meetings/workshops and other interventions are used to inform implementation. Reporting and evaluation is the last step of the implementation plan • Australian: Integration with national safety to help health services to meet specific requirements. To support clinicians to use the best available evidence, including clinical care standards, and to monitor and respond to unwarranted clinical variation. The clinical care standard was disseminated through the publication of editorials in the journals related to low back pain
Update	• UK: 3 years after publication and 2 years in special circumstances • Canadian: It is scheduled to be updated each year in the first 3 years after development • Australian: The standard will be updated but a specific time was not provided

*UK* United Kingdom

The clinical care standards provide additional quality statements for patients (specifying the type of care to be received), health care professionals (specifying the type of care to be delivered), and services (specifying resources needed for implementation). The UK clinical care standard also includes a statement directed to Commissioners [e.g., clinical commissioning groups and National Health Service (NHS) in England].

The UK and Canadian clinical care standards provide three types of quality indicators for monitoring the implementation process: structure indicators (i.e., aspects of the setting where health care is offered); process indicators (i.e., what care is offered by health care professionals and received by patients); and outcome indicators (i.e., result of the care implemented on patient’s health) [15]. The Australian clinical care standard provides structure and process indicators. The Canadian clinical care standard has 1 structure indicator, 10 process indicators, and 2 outcome indicators; the UK clinical care standard includes 7 structure indicators, 7 process indicators, and 4 outcome indicators; and the Australian clinical care standard includes 6 structural indicators and 6 process indicators.

### Consistent recommendations provided in the three clinical care standards

Table 2 provides the comparison between quality statements of the clinical care standards, and Table 3 provides the quality indicators in the clinical care standards. The supplemental Table 1 details the quality indicators proposed in the clinical care standards.

**Table 2:** Quality statements provided by the UK, Canadian, and Australian clinical care standards

Topic	Quality statement
Comprehensive assessment	• UK: Nil • Canadian: Provide a prompt comprehensive assessment for patients seeking primary care • Australian: The assessment of a patient with a new presentation of low back pain symptoms, with or without leg pain or other neurological symptoms, focuses on screening for specific and/or serious pathology and consideration of psychosocial factors. It includes a targeted history and physical examination, with a focused neurological examination when appropriate
Psychological assessment	• UK: Primary care services have an approach to risk stratification for new episodes • Canadian: Potential psychosocial risk factors for developing chronic pain are identified as yellow flags. Yellow flags may be identified through a set of questions or, if persists after education reassurance, supporting tools can be used • Australian: Early in each new presentation, a patient with low back pain, with or without leg pain or other neurological symptoms, is screened and assessed for psychosocial factors that may affect their recovery. This includes assessing their understanding of, and concerns about, diagnosis and pain, and the impact of pain on their life. The assessment is repeated at subsequent visits to measure progress
Diagnostic imaging	• UK: Do not have imaging requested by a non-specialist service unless serious underlying pathology is suspected • Canadian: Do not request diagnostic imaging tests unless they present with red flags that suggest serious pathological disease • Australian: Expectations of imaging and its limited role in diagnosing low back pain are discussed with a patient. Early and appropriate referral for imaging occurs when there are signs or symptoms of specific and/or serious pathology. The likelihood and significance of incidental findings are reported and discussed with the patient
Review and referral	• UK: Nil • Canadian: If the patient has unmanageable disabling back or leg pain (i.e., is unable to perform their usual daily activities), if their limitations from back pain are ongoing and substantial, or if their symptoms are worsened by physical activity and exercise, an appropriate referral should be made to a spine-focused provider (In “Clinical assessment statement”) • Australian: A patient with persisting or worsening symptoms, signs or function is reassessed at an early stage to determine the barriers to improvement. Referral for a multidisciplinary approach is considered. Specialist medical or surgical review is indicated for severe or progressive back or leg pain that is unresponsive to other therapy, progressive neurological deficits, or other signs of specific and/or serious pathology
Patient education and self-management	• UK: Provide advice and information to patients self-manage their condition • Canadian: Provide education and ongoing support for self-management that is tailored to people’s needs • Australian: A patient with low back pain is provided with information about their condition and receives targeted advice to increase their understanding, and address their concerns and expectations. Self-management is discussed with the patient because it will differ for each patient
Advice to stay active	• UK: Nil • Canadian: Encourage people to stay physically active by continuing to perform activities of daily living, with modifications if required • Australian: A patient with low back pain is encouraged to stay active and continue, or return to, usual activity, including work, as soon as possible or feasible
Psychological support	• UK: Nil • Canadian: Provide information and support for those who have psychosocial barriers to recovery (yellow flags) identified during their comprehensive assessment • Australian: A patient with low back pain is offered physical and/or psychological interventions based on their clinical and psychosocial assessment findings
Non-opioid analgesics	• UK: Do not prescribe paracetamol alone, anticonvulsants or antidepressants for low back without sciatica • Canadian: Provide information on risks and benefits of non-opioid analgesics to improve mobility and function for whose symptoms do not adequately improve with physical activity, education, reassurance, and self-management support • Australian: Anticonvulsants, benzodiazepines and antidepressants are avoided, because their risks often outweigh potential benefits, and there is evidence of limited effectiveness
Opioids	• UK: Do not prescribe opioids for chronic low back pain without sciatica • Canadian: Opioids should not be used routinely to treat acute low back pain. In some circumstances, it is reasonable to prescribe opioids at the lowest effective dose for a limited time if patients with severe pain and disability are unresponsive to non-pharmacological therapies and medications. (In “Pharmacological statement”) • Australian: Opioid analgesics are considered only in carefully selected patients, at the lowest dose for the shortest duration possible
Non-pharmacological treatment	• UK: Do not provide spinal injections except radiofrequency denervation for people who meet the criteria • Canadian: Provide information on the risks and benefits of additional non-pharmacological therapies to improve mobility and function for whose symptoms do not adequately improve with physical activity, education, reassurance, and self-management support • Australian: Physical intervention (i.e., heat wraps and massages) may be helpful as a part of treatment package including physical activity for a short period of time. Advise that physical activity and exercise therapy can help patients with an acute exacerbation of persistent or chronic low back pain

*UK* United Kingdom

**Table 3:** UK, Canadian and Australian indicators in the quality statements across clinical care standards

	Structure indicators			Process indicators			Outcome indicators		
Quality domains	UK	Canadian	Australian	UK	Canadian	Australian	UK	Canadian	Australian
Assessment and diagnosis									
Comprehensive assessment		Yes	Yes		Yes	Yes			
Risk stratification	Yes		Yes						
Diagnostic imaging	Yes		Yes	Yes	Yes	Yes			
Treatment									
Advice to self-manage the condition	Yes			Yes	Yes	Yes	Yes	Yes	
Advice to maintain usual activities			Yes		Yes				
Psychological support			Yes			Yes		Yes	
Non-opioids analgesics	Yes			Yes	Yes	Yes	Yes		
Opioids	Yes			Yes	Yes	Yes	Yes		
Non-pharmacological treatment	Yes			Yes	Yes				
Referral to a specialist			Yes		Yes				

*UK* United Kingdom

#### Diagnostic imaging

The three clinical care standards provided consistent recommendations in the statements related to diagnostic imaging (Table 2). All statements recommend that patients with low back pain should not receive lumbar spine imaging unless serious pathologies are suspected. However, the Canadian and Australian clinical care standards describe a set of “red flags” that suggest serious pathological disease and types of diagnostic imaging required, whereas the UK statement describes serious pathologies and refers the reader to other NICE guidelines for further guidance. The UK and Australian clinical care standards recommend explaining the reasons for not requesting imaging.

The clinical care standards suggest different indicators to monitor lumbar imaging requests. While the UK process indicator focuses on rates of inappropriate imaging, the Australian process indicator focuses on the proportion of appropriate imaging. In contrast, the Canadian process indicator focuses on documenting all low back pain imaging. None of the standards provides an indicator for underuse of lumbar imaging (i.e., failure to request imaging when indicated).

#### Patient education/advice and self-management

The three care standards provide statements recommending that patients should receive advice to self-manage low back pain (Table 2). The statements recommend that patients should be reassured about the condition’s benign nature and likely rapid resolution and be advised to quickly return to normal activities (including physical activity, exercise, and work). The UK and Australian standards provide supporting information and leaflets for patients and clinicians, while the Canadian statement recommends written and electronic tools as well as translation to relevant languages.

Staying physically active and returning to work is also recommended by all clinical care standards. The Canadian and Australian clinical care standards have a specific statement recommending that patients should avoid bed rest, stay active, gradually increase physical activity, and return to usual activities (Table 2). In contrast, the UK standard recommends staying active and returning to work in the self-management statement. Nevertheless, the main message is consistent across all clinical care standards.

There are some differences in the indicators from the clinical care standards. The three standards suggest generic indicators measuring the proportion of patients receiving education/advice to self-manage the condition. However, while the Canadian standard provides an outcome indicator to assess confidence in self-management using the Pain Self-Efficacy Scale [16], the UK standard recommends an indicator to assess patient satisfaction and the number of repeat GP appointments.

### Consistent recommendations provided by two clinical care standards

#### Comprehensive assessment

The Canadian and Australian clinical care standards have a quality statement recommending performing a prompt comprehensive assessment (Table 2). The comprehensive assessment (i.e., appointments within 1–3 days for urgent requests) should include outcome measurement, yellow and ‘red flags’ assessment, and referral for follow-up with their primary care provider if symptoms do not improve after 4 weeks. The Canadian standard also suggests some assessment tools, such as the Brief Pain Inventory-Short Form [17] and the Clinically Organized Relevant Exam (CORE) Back Tool [18].

Both clinical care standards differ in the process indicators related to comprehensive assessment. The Canadian indicators measure the number of days waiting for the comprehensive assessment and the proportion of patients referred to a spinal specialist due to specific reasons including “*symptoms that worsen with physical activity and exercise*” and “*unmanageable disabling back or leg pain*”. In contrast, the Australian indicator assesses the proportion of patients with findings of the assessment documented in their medical records.

#### Psychological support/interventions

The Canadian and Australian clinical care standards recommend offering psychological approaches for patients with acute low back pain (Table 2). The use of these approaches is recommended in both standards alongside other non-pharmacological approaches for patients with identified psychosocial barriers to recovery. The Canadian statement includes as examples psychological support/interventions, such as individual counselling and evidence-based treatment for mood disorders, whereas the Australian standard focuses on cognitive behavioural therapy, progressive relaxation, or mindfulness-based stress reduction.

Regarding the indicators, while the Canadian standard suggests an outcome indicator to measure the proportion of patients with identified psychological barriers who have been given information and support, the Australian standard intends to measure the proportion of people referred to physical and/or psychological services.

#### Review and referral of patients

The Canadian and Australian standards provide recommendations on reviewing and referring patients with persisting and worsening symptoms to a specialist. While the Australian standard provides a specific statement, the Canadian standard includes the recommendation in the “Clinical assessment” statement. Furthermore, the Australian standard recommends referring these patients to a multidisciplinary approach or a specialist and surgical review of those patients presenting severe or progressive back or leg pain that is unresponsive to other therapy, progressive neurological deficits, or other signs of specific and/or serious pathology. Regarding the quality indicator, while the Canadian standard intends to measure the proportion of patients referred to a specialist, the Australian standard focuses on the evidence of a policy for the review and referral of patients. Nevertheless, both standards provide consistent recommendations for the review and patients referral.

### Discrepancies across the three clinical care standards

#### Psychological assessment

The three clinical care standards differ in their recommendations related to psychological assessment. The UK and Australian standards provide an individual quality statement (Table 2), while the Canadian standard includes the recommendation in the comprehensive assessment statement. Although all standards recommend the use of supporting tools (e.g., STarT Back screening tool), the timing of its administration differs between them. In the UK and Australian standards, it is recommended to be applied in the first consultation, while the Canadian standard recommends its use only if yellow flags (assessed using questions from the CORE back tool [18]) persist after education and reassurance. Although all standards support the identification of patients with an increased risk of poor prognosis, they endorse different approaches at different stages in the episode of care.

#### Opioid analgesics

The clinical care standards differ in their recommendations on opioids. The UK statement provides a very clear message against opioid use for chronic low back pain (Table 2). The Canadian and Australian standards recommend opioids to a subset of patients with acute low back pain who still have severe pain and disability at the shortest and lowest dose possible. Moreover, while the Canadian standard also recommends opioids for those who have not responded to other treatments, the Australian standard recommends that the opioid status of the patient should also be considered.

All clinical care standards provide consistent indicators to measure the proportion of patients who are prescribed opioids, although they differ in the duration of symptoms. The UK indicator focuses on people with chronic low back pain, the Canadian indicator focuses on people with acute low back pain, and the Australian indicator does not specify the duration of symptoms.

#### Non-opioid analgesics

The standards provide different recommendations regarding non-opioid analgesics (Table 2). The Canadian statement encourages clinicians to provide information on the risks and benefits of non-opioid analgesics and recommends non-steroidal anti-inflammatories (NSAIDs) as the first pharmacological option if patients with acute low back pain do not improve with non-pharmacological therapy. In addition, the Canadian statement also recommends the use of muscle relaxants if patients do not respond to NSAIDs. In contrast, the UK and Australian statements focus on pharmacological treatment that should not be used for low back pain due to their limited effectiveness, including anticonvulsants and antidepressants. Moreover, the UK statement also does not recommend the use of paracetamol.

The three clinical care standards provide different indicators related to pharmacological options but are in line with the statements endorsed. While all standards provide indicators related to the use of medicines, the Canadian standard also suggests an indicator to measure if patients are receiving information on the risks and benefits of non-opioid analgesics. Moreover, the UK standard suggests an outcome indicator measuring the number of adverse events related to the use of medications.

#### Non-pharmacological therapies

The clinical care standards provide different recommendations on non-pharmacological treatments (Table 2). The Canadian standard recommends providing information on the risks and benefits of non-pharmacological options and suggests the use of massage, superficial heat, acupuncture, and manual therapy in combination with physical activity as appropriate non-pharmacological therapies. The Australian standard recommends physical approaches (e.g., heat wraps and massage) alongside to individualised physical activity, and psychological approaches. In contrast, only the UK standard provides a recommendation against the use of spinal injections with the exception of radiofrequency denervation that can be used for patients with chronic low back pain meeting specific criteria.

The process indicators related to the non-pharmacological options align with the statement provided by the standards. The Canadian and Australian indicators monitor the the proportion of patients receiving non-pharmacological options, although the Australian focus on those at risk of poor outcomes. The UK indicator estimates the amount of appropriate use of spinal injections according to the criteria for radiofrequency denervation. These criteria include, for example, reporting a pain intensity of at least five out of ten points in the visual analogue scale.

### Structure indicators across clinical care standards

All clinical care standards suggest structure indicators to measure the characteristics of the setting where the care will be implemented (Table 3). The UK standard suggests at least one structure indicator per statement, the Australian standard provides one structure indicator for each statement (except for the statement related to education/advice), and the Canadian standard provides only one structure indicator in the comprehensive assessment statement. While most UK structure indicators measure the evidence of local arrangements with health care professionals, the Australian standard focuses on a broader perspective with the implementation of policies for providing supporting tools, referral pathways, and training for implementation of the statements. Meanwhile, the structure indicator of the Canadian clinical care standard recommends measuring the availability of “*rapid access clinics*”.

### Data sources to collect quality indicators

The Canadian and UK clinical care standards suggest data sources for the indicators. Both standards suggest local practice data collection as data sources for most indicators. The UK standard provides examples of what data should be collected for measurement of the indicators, such as service protocols, patient notes, and prescribing audits. In addition to the local data collection, the Canadian standard also recommends the use of national databases for a few indicators, such as to estimate the number of patients with low back pain and the proportion of patients that receive a prescription of opioids. The only national data source suggested by the UK clinical care standard is the National Pain Audit, which collects data on patient satisfaction [19]. The Australian standard does not provide the data source, but it provides a link to the registration of the quality indicators in the Meta Online Registry (METEOR) which contains information for data collection and calculation.

## Discussion

### Summary of findings

The three clinical care standards provide consistent recommendations in the statements related to comprehensive assessment, imaging, patient education/advice, self-management of the condition, and review and patients referral. There is inconsistency in the quality statements related to psychological assessment, opioid prescription, non-opioid analgesics, and non-pharmacological therapies. We also found inconsistencies in the indicators suggested by the standards related to the number, type, and aspects of care assessed by the indicators.

### Possible explanations for the discrepancies observed between documents

The clinical care standards have focused on different target groups related to the duration of symptoms. While the Canadian and Australian standards provide recommendations for patients with acute low back pain, the UK standard provides recommendations regardless of the duration of symptoms. Discrepancies across the sets of quality statements may also be explained by the fact that each determined critical areas for improvement in their setting which may differ across geographical contexts.

### Challenges for implementation of quality statements

The first step in the implementation of the clinical care standards is to identify and provide support to key audiences noting that challenges to implementation may differ for different audiences as well as for different settings. For example, the proportion of patients with serious pathologies is likely to be higher in emergency departments compared to primary care settings [20]. Therefore, emergency staff may need a higher index of suspicion in ruling out serious pathologies. At the same time, time constraints and the busy environment of emergency departments might also mean that there is only time for standard provision of patient education and advice to self-manage their condition rather than tailoring it for the individual patient [21]. None of the standards included specific indicators to monitor the differentiation of inflammatory from non-inflammatory pain as this is likely not easily or reliably measured. However, both the Australian and Canadian standards recommended appropriate early referrals when needed to relevant healthcare providers including rheumatologists and an indicator to monitor that referral pathways to appropriate healthcare providers are in place. However, the implementation in primary health care would also have challenges, as it requires voluntary initiative from clinicians and would face similar barriers to the implementation of clinical practice guidelines such as the restriction of clinical judgment [22]. In addition, the implementation of clinical care standards would also require an appropriate “pre-implementation” stage, engaging with healthcare organisations to ensure the buy-in of health managers and department directors. Future studies should investigate the barriers to implementation of the clinical care standards and whether further strategies are needed for the implementation of quality statements in different settings.

Reducing unnecessary imaging for low back pain is consistently recommended across all clinical practice guidelines for non-specific low back pain [7]. Similarly, all clinical care standards consistently recommended avoiding imaging requests for non-specific low back pain. Nevertheless, imaging rates do not seem to have reduced over time; in fact, there is some evidence that complex imaging rates might have increased by about 50% from 1995 to 2015 [23]. Given that providing support tools is suggested as part of the implementation of clinical care standards, some strategies might be effective to reduce imaging rates, such as clinical decision support, targeted reminders, and audit and feedback [24]. Moreover, we need to test the scalability of these interventions to reduce imaging rates. For example, a recent clinical trial showed that individualised audits and feedback at a national level were able to reduce the requests for musculoskeletal diagnostic imaging tests when compared to no intervention [25]. Nevertheless, the implementation of the clinical care standards may help understand the effects on imaging requests in the target settings.

The clinical care standards differ in their recommendations related to opioid use. While the UK standard discourages its use, the Canadian and Australian standards recommend to only a subset of patients at the shortest and lowest dose possible. This aligns with the available evidence showing the limited efficacy of opioids for people with chronic and acute low back pain [26, 27]. Although several implementation strategies have been tested, multi-faceted solutions may be required to reduce the use of opioids. For example, a recent trial in emergency departments showed that a multi-faceted strategy to implement an evidence-based model of care for low back pain was able to reduce opioid use (OR 0.57; 95% CI 0.38 to 0.85) [28], and sustain the reduction over the long-term [29]. Therefore, a de-implementation strategy may be planned ahead for an effective implementation of the clinical care standards.

### Challenges for data collection of the quality indicators

All three clinical care standards suggest indicators in each statement to be collected by the target health care settings. Some information may be extracted locally from electronic health records across different settings, such as the proportion of patients receiving pharmacological treatment. However, other indicators suggested may require extra information than those provided in electronic health records such as documented discussions between patients and doctors about self-management. Although the UK standard suggests the use of clinical notes for data collection of these indicators, this method has conflicting validity in other fields [30, 31] and there is a lack of validity studies in the low back pain field. In addition, some indicators may need additional tools to be collected, such as the use of self-reported instruments. For example, the Canadian standard recommends the referral of patients to the spinal specialist considering a specific set of criteria in which pain intensity should be evaluated. Therefore, health care settings should bear in mind these factors to ensure appropriate data collection and, consequently, to evaluate the implementation of the clinical care standards.

The standards also propose some indicators with inconsistent definitions in the literature. The UK standard suggests measuring the number of adverse events related to the use of medicines, although the different terminology and coding process could render data collection difficult [32, 33]. Similarly, the UK and Australian indicators related to lumbar imaging include determining the appropriateness of imaging for low back pain. However, the high variance in the criterion adopted by guidelines to define imaging appropriateness due to different red flags with limited predictive value [34] might influence the data collection of these indicators. Therefore, these terms may need to be standardised before implementation in the health care settings to ensure appropriate data collection.

### Strengths and limitations of the review

We performed a comprehensive search in MEDLINE and on Google to identify all clinical care standards for low back pain. However, we could not exclude the possibility of missing a document, although this may be a limitation of any review. In addition, the included clinical care standards were developed in high-income countries. The implementation of these documents in low- and middle-income countries would require some adaptations or the elaboration of new documents considering their geographical and cultural context.

## Conclusion

The three standards provide consistent recommendations in the statements related to imaging and patient education/advice and self-management of the condition. However, they differ in the recommendations related to psychological assessment, opioid prescription, non-opioid analgesics, and non-pharmacological therapies. The Canadian and Australian standards agree on the recommendations related to comprehensive assessment, psychological support, and review and patients referral.

### Supplementary Information

Below is the link to the electronic supplementary material.

Supplementary file1 (DOCX 18 KB)

## Data Availability

Data are available upon reasonable request.

## References

[1] GBD (2017). Disease and Injury Incidence and Prevalence Collaborators (2018) Global, regional, and national incidence, prevalence, and years lived with disability for 354 diseases and injuries for 195 countries and territories, 1990–2017: a systematic analysis for the Global Burden of Disease Study 2017. Lancet.

[2] Ferreira G, Costa LM, Stein A (2019). Tackling low back pain in Brazil: a wake-up call. Braz J Phys Ther.

[3] Pierobon A, Villalba F, Ferreira G, Maher CG (2021). Insights into low back pain management in Argentina. Braz J Phys Ther.

[4] Dieleman JL, Cao J, Chapin A (2020). US Health Care Spending by Payer and Health Condition, 1996–2016. JAMA.

[5] Foster NE, Anema JR, Cherkin D (2018). Prevention and treatment of low back pain: evidence, challenges, and promising directions. The Lancet.

[6] Jenkins HJ, Downie AS, Maher CG (2018). Imaging for low back pain: is clinical use consistent with guidelines? A systematic review and meta-analysis. Spine J.

[7] Oliveira CB, Maher CG, Pinto RZ (2018). Clinical practice guidelines for the management of non-specific low back pain in primary care: an updated overview. Eur Spine J.

[8] Australian Commission on Safety and Quality in Health Care (2015) Clinical Care Standards FAQs Clinicians and Health Services. In. Australian Commission on Safety and Quality in Health Care, Australia

[9] National Institute for Health and Care Excellence (2016) Quality standards: process guide. https://www.nice.org.uk/Media/Default/Standards-and-indicators/Quality-standards/quality-standards-process-guide-update-2016.pdf. Accessed 30 October 2019. p. 20–21

[10] Health Quality Ontario (2017) Quality Standards: Process and Methods Guide. https://hqontario.ca/Portals/0/documents/evidence/Quality_Standards_Process_and_Methods_Guide--Oct_2017.pdf. Accessed 30 October, 2019. p. 14

[11] McGowan J, Straus S, Moher D (2020). Reporting scoping reviews—PRISMA ScR extension. J Clin Epidemiol.

[12] Health Quality Ontario (2019) Low back pain Care For Adults with Acute Low Back Pain. https://www.hqontario.ca/Portals/0/documents/evidence/quality-standards/qs-low-back-pain-quality-standard-en.pdf. Accessed 20 February 2019

[13] National Institute for Health and Care Excellence (2017) Low back pain and sciatica in over 16s (Quality standard [QS155]). https://www.nice.org.uk/guidance/qs155. Accessed 08 March 2019

[14] Australian Commission on Safety and Quality in Health Care (2022) Low Back Pain Clinical Care Standard. In. ACSQHC, Sydney

[15] Donabedian A (1988). The quality of care. How can it be assessed?. JAMA.

[16] Nicholas MK (2007). The pain self-efficacy questionnaire: Taking pain into account. Eur J Pain.

[17] Cleeland CS; The Pain Research Group (1991) The Brief Pain Inventory. In. MD Anderson Cancer Center website

[18] Centre for Effective Practice (2016) Clinically Organized Relevant Exam (CORE) back tool [Internet]. In. The Centre, Toronto (ON)

[19] The National Pain Audit 2012 In.

[20] Ferreira GE, Machado GC, Abdel Shaheed C (2019). Management of low back pain in Australian emergency departments. BMJ Qual Saf.

[21] Pun JKH, Matthiessen CMIM, Murray KA, Slade D (2015) Factors affecting communication in emergency departments: doctors and nurses' perceptions of communication in a trilingual ED in Hong Kong. Int J Emerg 8(1):48–48. 10.1186/s12245-015-0095-y 10.1186/s12245-015-0095-y PMC4678128 26667242

[22] Slade SC, Kent P, Patel S, Bucknall T, Buchbinder R (2016). Barriers to Primary Care Clinician Adherence to Clinical Guidelines for the Management of Low Back Pain: A Systematic Review and Metasynthesis of Qualitative Studies. Clin J Pain.

[23] Downie A, Hancock M, Jenkins H (2019). How common is imaging for low back pain in primary and emergency care? Systematic review and meta-analysis of over 4 million imaging requests across 21 years. Br J Sports Med.

[24] Jenkins HJ, Hancock MJ, French SD (2015). Effectiveness of interventions designed to reduce the use of imaging for low-back pain: a systematic review. CMAJ.

[25] O’Connor DA, Glasziou P, Maher CG (2022). Effect of an Individualized Audit and Feedback Intervention on Rates of Musculoskeletal Diagnostic Imaging Requests by Australian General Practitioners: A Randomized Clinical Trial. JAMA.

[26] Abdel Shaheed C, Maher CG, Williams KA, Day R, McLachlan AJ (2016). Efficacy, Tolerability, and Dose-Dependent Effects of Opioid Analgesics for Low Back Pain: A Systematic Review and Meta-analysis. JAMA Intern Med.

[27] Jones CMP, Day RO, Koes BW (2023). Opioid analgesia for acute low back pain and neck pain (the OPAL trial): a randomised placebo-controlled trial. The Lancet.

[28] Coombs DM, Machado GC, Richards B (2021). Effectiveness of a multifaceted intervention to improve emergency department care of low back pain: a stepped-wedge, cluster-randomised trial. BMJ Qual Saf.

[29] Jones CM, Coombs D, Lin CC (2023). Implementation of a model of care for low back pain produces sustained reduction in opioid use in emergency departments. Emerg Med J.

[30] Parsons A, McCullough C, Wang J, Shih S (2012). Validity of electronic health record-derived quality measurement for performance monitoring. J Am Med Inform Assoc.

[31] Bierman JA, Hufmeyer KK, Liss DT, Weaver AC, Heiman HL (2017). Promoting Responsible Electronic Documentation: Validity Evidence for a Checklist to Assess Progress Notes in the Electronic Health Record. Teach Learn Med.

[32] Musy SN, Ausserhofer D, Schwendimann R (2018). Trigger Tool-Based Automated Adverse Event Detection in Electronic Health Records: Systematic Review. J Med Internet Res.

[33] Phillips R, Hazell L, Sauzet O, Cornelius V (2019). Analysis and reporting of adverse events in randomised controlled trials: a review. BMJ Open.

[34] Yates M, Oliveira CB, Galloway JB, Maher CG (2020). Defining and measuring imaging appropriateness in low back pain studies: a scoping review. Eur Spine J.

