# Supplementary material for: Clinical care standards for the management of low back pain: a scoping review
Source: Rheumatol Int. 2024 Feb 29;44(7):1197–207. doi: 10.1007/s00296-024-05543-2 (PMC11178557; doi:10.1007/s00296-024-05543-2)
Supplement: Supplementary file 1 — Supplementary file1 (DOCX 18 KB) [file 296_2024_5543_MOESM1_ESM.docx]

| ***Appendix 1. NICE, Canadian and Australian indicators in the quality statements across clinical care standards*** | | | | | | | | | | |  |
| --- | --- | --- | --- | --- | --- | --- | --- | --- | --- | --- | --- |
|  | **Structure indicators** | | | **Process indicators** | | | **Outcome indicators** | | | |  |
| ***Quality statements*** | ***NICE*** | ***Canadian*** | ***Australian*** | ***NICE*** | ***Canadian*** | ***Australian*** | ***NICE*** | ***Canadian*** | ***Australian*** |  |  |
| *Assessment and diagnosis* |  |  |  |  |  |  |  |  |  |  |  |
| Comprehensive assessment |  | Local availability of rapid access clinics | Evidence of a locally approved low back pain assessment protocol |  | - Number of days waiting for assessment  - Percentage of people referred to a spine-focused provider | Proportion of patients with acute low back pain underlying pathology documented in their medical record. |  |  |  |  |  |
| Risk stratification | Locally defined approach and systems in place to make staff aware |  | Evidence of a locally approved policy to ensure that patients are screened for psychosocial factors early in each new presentation. |  |  |  |  |  |  |  |  |
| Diagnostic imaging | - Local arrangements for people to be referred for specialist opinion.  - Local protocols outlining serious underlying pathology |  | Evidence of a locally approved policy to ensure the appropriate use of imaging for low back pain. | Proportion of people who had diagnostic imaging requested when no serious underlying pathology is suspected | Proportion of people who undergo diagnostic imaging | Proportion of patients with a new episode of low back pain referred for imaging for whom an appropriate indication for imaging is documented in the medical record. |  |  |  |  |  |
| Review and referral |  |  | Evidence of a locally approved policy that defines the process for review and referral of patients with low back pain. |  | Percentage of people with acute low back pain who are referred to a spine-focused provider for any of the following:  * Unmanageable disabling back or leg pain  * Limitations from back pain that are ongoing and substantial  * Symptoms that worsen with physical activity and exercise |  |  |  |  |  |  |
|  |  |  |  |  |  |  |  |  |  |  |  |
| *Treatment* |  |  |  |  |  |  |  |  |  |  |  |
| Advice to self-manage the condition | Local arrangements to ensure that staff have access to information and the knowledge needed to signpost to other services |  |  | Proportion of people who are given advice and information to self-manage their condition. | Percentage of people who receive education and ongoing support for self-management | Proportion of patients with low back pain who have documented discussions in their medical record about both self-management strategies and staying active by continuing usual activities. | - Number of repeat GP appointments  - Levels of satisfaction | - Percentage of people who report feeling confident about self-managing the condition |  |  |  |
| Advice to maintain usual activities |  |  | Evidence of local arrangements to ensure that patients are provided with information, advice and reassurance. |  | - Percentage of people who have documented discussions about staying physically active  - Percentage who have documented discussions about continuing work or returning to work  - Number of days from take a leave of absence from work to when they return to work |  |  |  |  |  |  |
| Psychological support |  |  | Evidence of a locally approved policy that specifies the referral pathways to clinicians who provide appropriate psychological therapies. |  |  | Proportion of patients with low back pain at risk of poor outcomes who were referred to physical and/or psychological clinical services. |  | Percentage of people with identified psychosocial barriers to recovery who has given information and support to manage them |  |  |  |
| Non-opioids analgesics | Local arrangements to ensure that no GP prescriptions include paracetamol alone, anticonvulsants or antidepressants |  |  | - Proportion of people who are given anticonvulsants  - Proportion of people who are given antidepressants  - Proportion of people who are given paracetamol | Percentage of people whose symptoms are not improving with nonpharmacological therapies who are given information on the risks and benefits of nonopioid analgesics | Proportion of patients who received an anticonvulsant | Number of medicines-related adverse events |  |  |  |  |
| Opioids | Local arrangements to ensure that no GP prescriptions include opioids to treat chronic low back pain |  |  | Proportion of people who are given opioids to treat chronic low back pain | Percentage of people with acute low back pain who are prescribed an opioid medication | Proportion of patients with low back pain who received an opioid analgesic | Number of opioids-related adverse events for chronic low back pain without sciatica |  |  |  |  |
| Non-pharmacological treatment | Local arrangements to ensure that spinal injections are not given, except for radiofrequency denervation for people who meet the criteria. |  |  | Proportion of people who have spinal injections who meet the criteria for radiofrequency denervation. | Percentage of people whose symptoms do not adequately improve with physical activity, education, reassurance, and self-management support who receive one or more additional nonpharmacological therapies |  |  |  |  |  |  |
| Abbreviations: GP, General Practitioner; NICE, National Institute for Health and Care Excellence. | | | | | | | | | | | |
